# A Dataset for Addressing Patient’s Information Needs related to Clinical Course of Hospitalization

**DOI:** 10.1038/s41597-026-06639-z

**Published:** 2026-02-25

**Authors:** Sarvesh Soni, Dina Demner-Fushman

**Affiliations:** Division of Intramural Research, National Library of Medicine, National Institutes of Health, Bethesda, MD USA

**Keywords:** Patient education, Computational science, Health services

## Abstract

Patient’s unique information needs about their hospitalization can be addressed using clinical evidence from electronic health records (EHRs) and artificial intelligence (AI). However, robust datasets to assess the factuality and relevance of AI-generated responses are lacking and, to our knowledge, none capture patient information needs in the context of their EHRs. To address this gap, we introduce ArchEHR-QA, an expert-annotated dataset of 134 cases from intensive care unit and emergency department settings. Cases comprise patient questions from public health forums, clinician-interpreted versions, relevant clinical note excerpts with sentence-level relevance annotations, and clinician-authored answers. To establish benchmarks for grounded EHR question answering (QA), we evaluated three open-weight large language models (Llama 4, Llama 3, and Mixtral) across three prompting strategies. We assessed performance on two dimensions: Factuality (overlap between cited and ground truth evidence) and Relevance (similarity to reference answers).

## Background & Summary

Question answering (QA) is an organic way to interact with complex information systems such as electronic health records (EHRs)^1^, where a QA system responds to user questions with exact answers. The major focus of existing EHR QA work has been on addressing clinician information needs^2^, with datasets for system development and evaluation largely prioritizing these requirements^3^. However, with the increasing patient-involvement in their care^4–8^, there is a need for targeted EHR QA research that incorporates the unique needs of patients from their health records^9,10^. To this end, datasets play an important role in developing and evaluating tailored artificial intelligence (AI) systems and, thus, the datasets must be representative of the target end-user’s needs^11^, i.e., patients.

Despite growing interest in patient-centered care, most work on addressing the health information needs of patients uses general health resources^12^. This disconnect often results in systems that fail to incorporate the context that prompts the patients’ questions, i.e., EHRs, contributing to non-patient-centered solutions^13^. EHRs document important clinical evidence about the care provided to patients. However, the rationale for drafting these documents often differs from the primary reasons patients access them^14^. This necessitates building assistive technologies to contextualize EHR information to address patients’ information needs.

Moreover, the volume of patient requests for medical information through patient portals is rising, contributing to desktop medicine and increasing clinician burden^15^. Most existing studies on automated responses to patient messages do not incorporate critical contextual information from EHRs^16–20^. Among studies that use EHR content, none provide comprehensive evaluations of how effectively the generated responses leverage this clinical context^21,22^.

Grounding is crucial in AI applications in medicine, as it ensures that AI models are anchored to accurate, contextually relevant, real-world clinical data. This is particularly important when the intended audience lacks clinical expertise^23^. To effectively design and evaluate grounded QA systems, a representative dataset and evaluation framework are essential^24^.

In this work, we propose a benchmark dataset, ArchEHR-QA, to evaluate grounding capabilities of models for responding to patient-initiated queries. The dataset consists of patient-initiated questions posted in public domain, the corresponding clinician-interpreted questions, the excerpts of the EHRs annotated at the sentence-level with relevance to the question, and clinician-generated free-text answers to the questions grounded with EHR sentences. We collect true patient health information needs expressed in real-world health forum messages, we then align the messages to publicly accessible real EHRs. To our knowledge, this is the first public dataset that encapsulates patient questions and relevant clinical evidence from EHRs. We further provide an evaluation framework to assess two critical aspects of a grounded EHR QA system: does it identify relevant information from given clinical evidence and does it use this information in responding to user queries.

## Methods

### Data

**Fig. 1:**
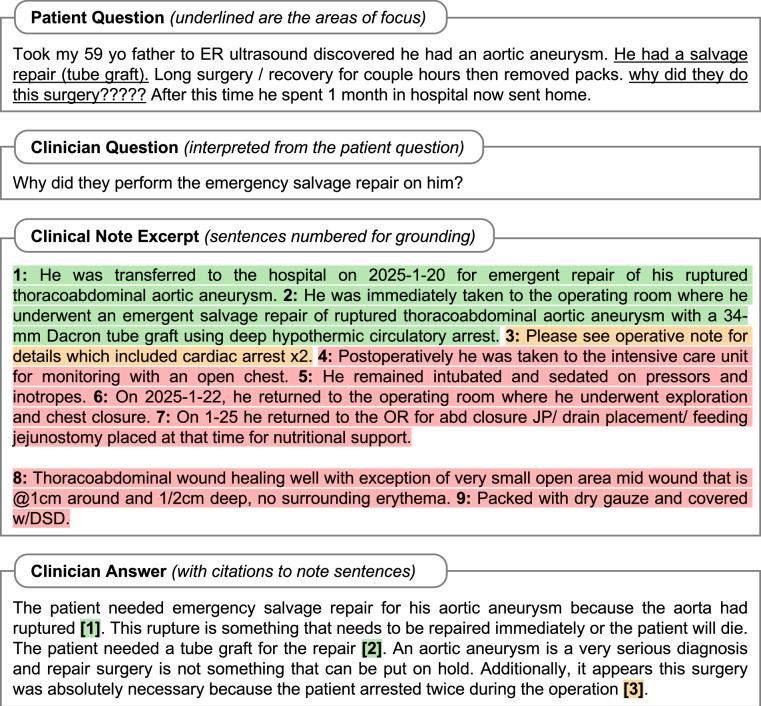
Example annotated case from the dataset. In Clinical Note Excerpt, sentences with ID 1 and 2 are essential, 3 is supplementary, and others are not-relevant. In Clinician Answer, some sentences may be unsupported.

There is no public resource containing both patient-initiated questions and their EHRs. Thus, we created ArchEHR-QA by aligning two different publicly available resources for patient questions and EHRs (Fig. 1). To incorporate true patient information needs, we derive questions using public health forums, one of the most popular internet venues for patients to seek information where patients pose their information needs as discussion posts^25,26^. Specifically, we use the publicly available health forum discussions collected from HealthCareMagic.com and released as part of an existing work^27^, which can be accessed through GitHub at https://github.com/Kent0n-Li/ChatDoctor. To associate relevant medical context to the questions, we find clinical evidence using discharge summaries from publicly accessible EHR data. Specifically, we source discharge summaries from MIMIC-III^28^ and MIMIC-IV^29^ databases, both of which contain deidentified patient records and can be accessed via PhysioNet^30,31^.

### Alignment

Health forum posts were filtered to reflect scenarios in which patients had recently been discharged from the hospital—a common context for post-encounter online information seeking^32^. The posts were filtered using keywords such as “admission” (“admit”, “admitted”, “admitting”, “admits”), “emergency room” (“er”, “ed”, “emergency department”, “emergency dept”, “emergency”), and “ICU” (“ccu”, “intensive care”, “critical care”, “care unit”). All filtered posts were manually reviewed by SS (clinical informaticist) and DDF (MD and computational linguist). For each reviewed forum post, a set of candidate relevant discharge summaries (notes) were retrieved from the EHR. Due to the differences in vocabulary between patient-authored posts and clinician-authored notes (e.g., *“pelvic”* vs *“inferior perineal”*), we augmented the text in both sources with standardized terminology. For forum posts, we appended ICD code descriptions automatically generated by prompting the Llama 3.3 70B^33^ large language model (LLM). For clinical notes, we appended the corresponding ICD code descriptions associated with the patient’s admission (available as part of the structured data in the MIMIC databases). The expanded posts were used to retrieve candidate notes using BM25 algorithm^34^. The top 10 notes for each post were manually reviewed by SS and DDF to select only the ones that may be used as evidence to satisfy the information needs expressed in the forum post. For each selected note, the corresponding forum post was minimally modified to match the surrounding details (e.g., to ensure its alignment with the note. We never modified the underlying information needs in the post or the associated note content. Annotation guidelines are provided under Supplementary Information.

### Question and answer creation

**Fig. 2:**
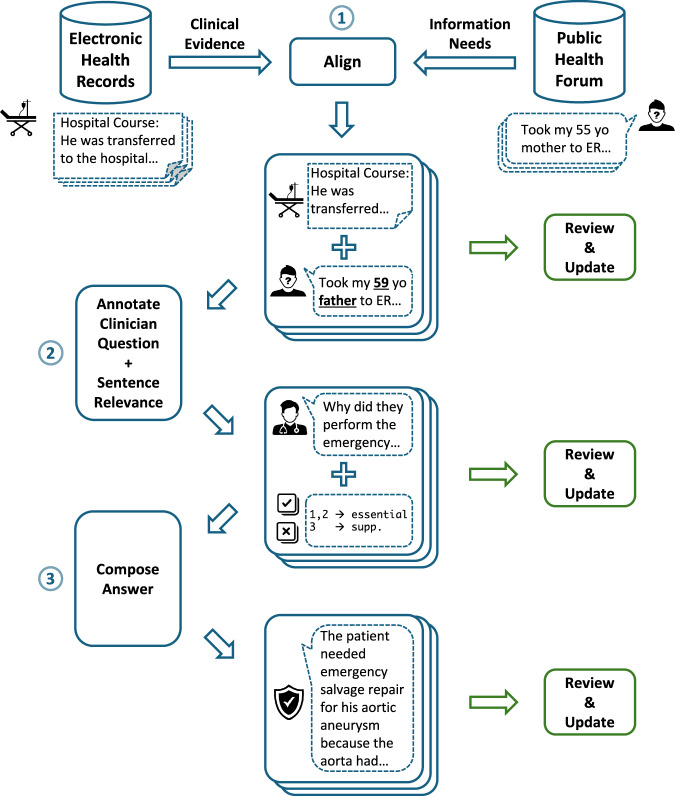
Dataset creation workflow. (**1**) Patient information needs identified from a public health discussion forum are aligned with clinical evidence from an electronic health record database to produce pairs of patient-posed questions and associated clinical notes. (**2**) Clinician-interpreted questions are created from patient questions and sentence-level relevance labels are annotated in the note. (**3**) Clinicians compose natural language answers considering the notes. Annotations are reviewed and updated at each stage to ensure high quality.

For each aligned forum post-note pair, SS, DDF, and a licensed clinician annotated the query-related focus areas in the post and created a clinician’s version of the patient’s query—this is a question that a clinician would interpret and respond to (Fig. 2). Further, each sentence in the clinical note was manually annotated with a *“relevance”* label, indicating its importance in answering the question: *“essential”* (must be used), *“supplementary”* (provides support but not necessary), or *“not-relevant”*. Due to the considerable length of the notes, we manually curated *note excerpts* by excluding paragraphs composed entirely of sentences deemed *“not-relevant”*, while retaining those that provided important contextual information (e.g., section headers such as *“Brief Hospital Course”*). Lastly, we tasked licensed clinicians (hired through Centaur Labs) to review and update the relevance labels for the note sentences and compose 75-word (approximately 5-sentence) *ground truth answers* to the questions. Furthermore, we annotated each case with clinical specialties (e.g., cardiology, neurology, pulmonology) for potential downstream use.

### Annotation agreement

All annotations were reviewed and revised as necessary by a second annotator. Alignment and sentence labeling was performed by the authors and reviewed by a licensed clinician, who revised the annotations as needed and authored an answer. Lastly, the clinician-written answer was reviewed by DDF, revising as needed. We report the annotation agreements for all stages.

In the first round of sentence relevance annotations review, clinicians agreed with the existing labels 77.1% (2653/3450) of the time, with the most common edits being changes from essential to supplementary (39.9% [318/797]) or to not-relevant (26.6% [212/797]). In the second round, the reviewer’s (DDF) agreement with the clinician-modified labels was 84.7% (2874/3450), with the predominant edit again being from essential to supplementary (56.1% [323/576]). Furthermore, the reviewer (DDF) retained 81.3% of the clinician-written answers, calculated as the proportion of word lemmas retained from the original answers while ignoring punctuations and stop words.

## Data Records

The proposed dataset, ArchEHR-QA^35^, is publicly accessible through PhysioNet^36^ at 10.13026/zzax-sy62. Components of the dataset, including patient questions, clinician questions, and clinical note excerpts, are provided in XML format. Additionally, sentence relevance keys and clinician answers are available in JSON format. The dataset is divided into development and test sets, with the test set comprising 100 cases (80 from the ICU and 20 from the ED). The PhysioNet repository includes detailed information on the dataset’s contents, including the structures of the XML and JSON files.

## Technical Validation

### Dataset analysis

The proposed dataset, ArchEHR-QA, contains a total of 134 patient cases—104 using intensive care unit (ICU) notes (sourced from the MIMIC-III database) and 30 using emergency department (ED) notes (sourced from MIMIC-IV). A sample annotated case is illustrated in Fig. 1, while Tables 1 and 2 provide detailed patient demographics and case statistics, respectively. The average length of patient narratives is 91.3 words (with a standard deviation [SD] of 56.9) while the clinician questions are 10.5 words (SD 3.6) long. The clinician-written answers have an average of 72.6 (SD 3.4) words. The note excerpts contain a mean of 25.7 sentences, 25.7% (6.6) of which are essential and 20.1% (5.2) are supplementary. The mean length of ICU note excerpts exceeds that of ED note excerpts, likely reflecting greater clinical complexity of ICU cases. This is also suggested by the prevalence of ICD codes in each setting, as presented in Table 3. ICU diagnoses predominantly involve heart, kidney, and respiratory failure, which often require intensive and multidisciplinary management, whereas ED diagnoses more often include single-system, episodic, or behavioral conditions such as asthma, apnea, and nicotine dependence. Table 4 presents the distribution of clinical specialties in the dataset. The most prevalent specialties among the cases are Cardiology, Neurology, Pulmonology, Infectious Diseases, and Gastroenterology. There is also a wide range of other specialties, each with fewer cases, forming a long tail distribution.

**Table 1:** Descriptive statistics of the patient population in the dataset.

Category	Value	ICU (N = 104)	ED (N = 30)	All (N = 134)
**Race or Ethnicity**	White	74 (71.2%)	17 (56.7%)	91 (67.9%)
	Black/African American	13 (12.5%)	7 (23.3%)	20 (14.9%)
	Hispanic/Latino	4 (3.8%)	2 (6.7%)	6 (4.5%)
	Asian	3 (2.9%)	2 (6.7%)	5 (3.7%)
	Other	1 (1.0%)	1 (3.3%)	2 (1.5%)
	Unknown/Declined	9 (8.7%)	1 (3.3%)	10 (7.5%)
**Sex**	Male	58 (55.8%)	10 (33.3%)	68 (50.7%)
	Female	46 (44.2%)	20 (66.7%)	66 (49.3%)
**Age Group**	0–30	8 (7.7%)	2 (6.7%)	10 (7.5%)
	31–40	5 (4.8%)	7 (23.3%)	12 (9.0%)
	41–50	13 (12.5%)	3 (10.0%)	16 (11.9%)
	51–60	20 (19.2%)	6 (20.0%)	26 (19.4%)
	61–70	25 (24.0%)	8 (26.7%)	33 (24.6%)
	71–80	16 (15.4%)	2 (6.7%)	18 (13.4%)
	81–89	15 (14.4%)	1 (3.3%)	16 (11.9%)
	90+	2 (1.9%)	1 (3.3%)	3 (2.2%)

ICU: Intensive Care Unit, ED: Emergency Department. Population statistics are in *count (proportion)* format.

**Table 2:** Descriptive statistics of the cases in the dataset.

Category	Value	ICU (N = 104)	ED (N = 30)	All (N = 134)
**Patient Narrative Word Count**	Mean	90.2	94.8	91.3
	Median	72.0	90.5	76.5
	Std Dev	61.7	36.6	56.9
	Min	33	54	33
	Max	440	192	440
**Clinician Question Word Count**	Mean	10.6	10.2	10.5
	Median	10.0	9.0	10.0
	Std Dev	3.6	3.8	3.6
	Min	3	4	3
	Max	21	21	21
**Answer Word Count**	Mean	72.6	72.3	72.6
	Median	73.0	73.5	73.0
	Std Dev	3.4	3.4	3.4
	Min	55	61	55
	Max	78	75	78
**Note Excerpt Word Count**	Mean	410.2	280.7	381.2
	Median	383.5	223.0	351.5
	Std Dev	200.1	196.8	205.9
	Min	107	76	76
	Max	1028	868	1028
**Mean Note Sentences Count**	All	27.6	19.3	25.7
	Essential	7.0 (25.5%)	5.2 (26.7%)	6.6 (25.7%)
	Supplementary	5.9 (21.4%)	2.6 (13.4%)	5.2 (20.1%)
	Not Required	14.7 (53.1%)	11.6 (59.8%)	14.0 (54.3%)

ICU: Intensive Care Unit, ED: Emergency Department. Mean values are in *count (proportion)* format.

**Table 3:** The top 10 ICD codes present in the dataset, stratified by the different clinical settings.

Setting	Value	ICU (N = 104)	ED (N = 30)	All (N = 134)
**ICU**	4019 - Unspecified essential hypertension	39 (37.5%)	3 (10.0%)	42 (31.3%)
	4280 - Congestive heart failure, unspecified	34 (32.7%)	0 (0.0%)	34 (25.4%)
	42731 - Atrial fibrillation	27 (26.0%)	0 (0.0%)	27 (20.1%)
	5849 - Acute kidney failure, unspecified	26 (25.0%)	3 (10.0%)	29 (21.6%)
	51881 - Acute respiratory failure	26 (25.0%)	0 (0.0%)	26 (19.4%)
	5990 - Urinary tract infection, site not specified	23 (22.1%)	0 (0.0%)	23 (17.2%)
	2724 - Other and unspecified hyperlipidemia	22 (21.2%)	2 (6.7%)	24 (17.9%)
	41401 - Coronary atherosclerosis of native coronary artery	22 (21.2%)	2 (6.7%)	24 (17.9%)
	486 - Pneumonia, organism unspecified	19 (18.3%)	0 (0.0%)	19 (14.2%)
	2761 - Hyposmolality and/or hyponatremia	18 (17.3%)	1 (3.3%)	19 (14.2%)
**ED**	K219 - Gastro-esophageal reflux disease without esophagitis	0 (0.0%)	8 (26.7%)	8 (6.0%)
	J45909 - Unspecified asthma, uncomplicated	0 (0.0%)	7 (23.3%)	7 (5.2%)
	G4733 - Obstructive sleep apnea (adult) (pediatric)	0 (0.0%)	6 (20.0%)	6 (4.5%)
	Z87891 - Personal history of nicotine dependence	0 (0.0%)	6 (20.0%)	6 (4.5%)
	I10 - Essential (primary) hypertension	0 (0.0%)	6 (20.0%)	6 (4.5%)
	F419 - Anxiety disorder, unspecified	0 (0.0%)	6 (20.0%)	6 (4.5%)
	F329 - Major depressive disorder, single episode, unspecified	0 (0.0%)	5 (16.7%)	5 (3.7%)
	D649 - Anemia, unspecified	0 (0.0%)	4 (13.3%)	4 (3.0%)
	F17210 - Nicotine dependence, cigarettes, uncomplicated	0 (0.0%)	4 (13.3%)	4 (3.0%)
	E785 - Hyperlipidemia, unspecified	0 (0.0%)	4 (13.3%)	4 (3.0%)

ICU: Intensive Care Unit, ED: Emergency Department. Population statistics are in *count (proportion)* format.

**Table 4:** Clinical specialties of the cases in the dataset, stratified by the different clinical settings.

Clinical Specialty	ICU (N = 104)	ED (N = 30)	All (N = 134)
Cardiology	24 (23.1%)	2 (6.7%)	26 (19.4%)
Neurology	18 (17.3%)	7 (23.3%)	25 (18.7%)
Pulmonology	15 (14.4%)	3 (10.0%)	18 (13.4%)
Infectious Diseases	11 (10.6%)	3 (10.0%)	14 (10.4%)
Gastroenterology	7 (6.7%)	4 (13.3%)	11 (8.2%)
Cardiovascular	8 (7.7%)	0 (0.0%)	8 (6.0%)
Hematology	5 (4.8%)	1 (3.3%)	6 (4.5%)
Oncology	5 (4.8%)	1 (3.3%)	6 (4.5%)
Hepatology	5 (4.8%)	0 (0.0%)	5 (3.7%)
Nephrology	5 (4.8%)	0 (0.0%)	5 (3.7%)
Traumatology	3 (2.9%)	2 (6.7%)	5 (3.7%)
Pain Management	1 (1.0%)	3 (10.0%)	4 (3.0%)
Psychiatry	2 (1.9%)	2 (6.7%)	4 (3.0%)
Rehabilitation	1 (1.0%)	3 (10.0%)	4 (3.0%)
Urology	3 (2.9%)	1 (3.3%)	4 (3.0%)
Endocrinology	3 (2.9%)	0 (0.0%)	3 (2.2%)
Immunology	2 (1.9%)	0 (0.0%)	2 (1.5%)
Internal Medicine	1 (1.0%)	1 (3.3%)	2 (1.5%)
Obstetrics	1 (1.0%)	1 (3.3%)	2 (1.5%)
Toxicology	2 (1.9%)	0 (0.0%)	2 (1.5%)
Genetics	1 (1.0%)	0 (0.0%)	1 (0.7%)
Gynecology	0 (0.0%)	1 (3.3%)	1 (0.7%)
Neuropsychology	1 (1.0%)	0 (0.0%)	1 (0.7%)
Neurosurgery	1 (1.0%)	0 (0.0%)	1 (0.7%)
Rheumatology	0 (0.0%)	1 (3.3%)	1 (0.7%)

ICU: Intensive Care Unit, ED: Emergency Department. Values are in *count (proportion)* format.

### Benchmarking

We performed benchmarking experiments for the task of automatically generating an answer to the input question (using both patient and clinician versions) with citations to the specific sentences in the input note excerpt. Based on existing literature^37,38^, we restricted the output answer length to 75 words (or approximately 5 sentences).

#### Evaluation framework

**Fig. 3:**
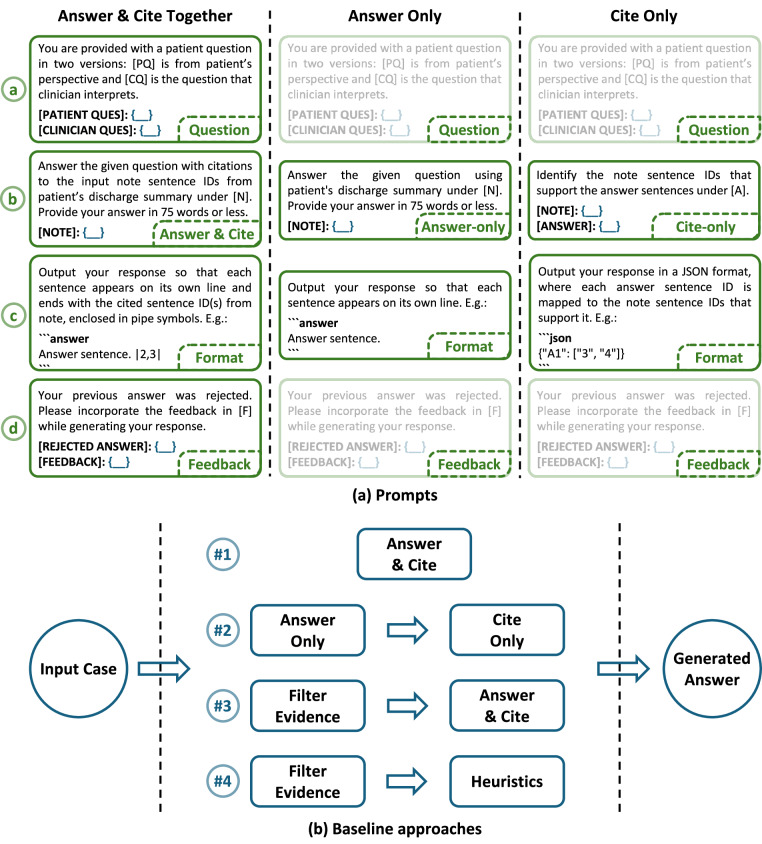
Overview of baseline approaches for the task of generating answers with citations. *(A) Prompt structures:* Each prompt is organized into four sections: (**a**) Input questions; (**b**) Instructions for the specific task component—*“Answer & Cite”*, *“Answer-only”*, or *“Cite-only”*; (**c**) Desired response format; and (**d**) Feedback from prior iterations, if available. Each prompt is executed up to five times to handle errors, after which the final response is output. *(B) Baseline approaches:* Four prompting approaches are evaluated: (1) *“Together”*—generating the answer and citations together; (2) *“Answer First”*—generating the answer first, then citations; (3) *“Evidence First”*—providing the model with filtered evidence sentences (based on cosine similarity to the questions); and (4) *“Heuristics”*—using evidence filtering followed by heuristic selection of supporting sentences until a word limit is reached.

The baseline approaches include a heuristic method that selects the most semantically similar note sentences using cosine similarity over MiniLM semantic embeddings^39^ and restricts the response to the top-ranked sentences within the word limit (Fig. 3). In addition to the heuristic baseline, we evaluated three prompting strategies using LLMs, each reflecting a distinct approach to citation integration: (1) generating answers with citations embedded directly in the output, (2) generating answers first followed by the addition of citations in a subsequent step, and (3) generating answers using only a specified set of citations as input. These strategies were designed to evaluate how citation context and ordering influence the model’s ability to produce factual and relevant responses.

All prompts are structured into four components: (a) an *input section* containing the patient and clinician questions; (b) *task-specific instructions*, which defined the objective of the prompt (e.g., for the *“Answer & Cite”* task, instructions explicitly directed the model to generate a response with inline citations); (c) *formatting instructions*, which specified the required output structure (e.g., for the *“Answer & Cite”* task, responses were to be formatted in markdown, with each answer sentence presented on a separate line followed by the corresponding citations); and (d) a *feedback section* providing error-specific guidance based on prior output. During prompt development, we observed several correctable errors, such as failing to include citations or format content on separate lines. To address these issues, we implemented an iterative prompting strategy, retrying each prompt up to five times. At each iteration, the model received programmatically generated feedback tailored to the specific formatting or content errors identified in the preceding response.

We benchmarked the zero-shot performance of three popular LLMs on ArchEHR-QA: Llama 3.3 70B^33^, Llama 4 17B 16E^40^, and Mixtral 8x22B^41^. These models represent top-tier performance among open-weight LLMs that can be run locally. The Llama models were developed by Meta AI, while the Mixtral model was released by Mistral AI. Llama 3.3 employs a standard transformer architecture, whereas Llama 4 and Mixtral leverage a mixture-of-experts (MoE) design to improve computational efficiency and performance.

#### Metrics

System-generated responses are evaluated along two dimensions: *Factuality* (use of clinical evidence for grounding) and Relevance (similarity to the ground-truth answer).

*Factuality* is measured using an F1 Score between the sentences cited as evidence in the system-generated answer (which are treated as predicted essential note sentences) and the ground truth relevance labels for note sentences. We define two versions of *Factuality: “Essential-only”* and *“Essential + Supplementary”*. In the *“Essential-only”* definition, only sentences labeled as essential in the ground truth count as positives. In the *“Essential + Supplementary”* version, ground truth sentences labeled as either essential or supplementary are counted as positives (penalizing the system for failing to cite either, but not for including supplementary ones). We report *Factuality* both at the *macro* level (averaging per-case F1 scores) and the *micro* level (aggregating true positives, false positives, and false negatives across all cases). We designate the essential-only micro F1 Score as *Overall Factuality* and use it to calculate the *Overall Score*, as it captures aggregate performance across all instances focusing on the most important note sentences.

*Relevance* is evaluated by comparing the generated answer text to a clinician-authored reference answer using text- and semantics-based metrics: BLEU^42^, ROUGE^43^, SARI^44^, BERTScore^45^, AlignScore^46^, and MEDCON^47^. Each metric is normalized, and *Overall Relevance* is computed as the mean of these normalized scores. We compute another version of relevance scores by treating the set of essential ground truth sentences (together with the original question) as the reference. This alternative provides a feasible and scalable approximation for evaluating answer quality in the absence of human-written answers.

#### Findings

Tables 5, 6, 7, and 8 summarize the benchmarking results on a held-out test set of 100 cases (80 from ICU and 20 from ED). The remaining 34 cases were used exclusively for the development of prompts. All the model-based approaches performed the best for *Answer First* variation, followed by *Together* and then *Evidence First* across most evaluation metrics, including the *Overall Factuality* and *Relevance*. Llama 4 consistently achieved superior *Factuality F1* scores across approaches with the best *Overall Factuality* (essential-only micro F1) of 51.8%, followed by Mixtral and Llama 3. In contrast, for *Relevance*, Mixtral outperformed the other models in most instances, achieving the best *Overall Relevance* of 33.8% (compared against human-authored answers), followed by Llama 3 and Llama 4.

**Table 5:** Factuality metrics results from benchmarking experiments.

Approach	Model	Essential-only						Essential + Supplementary					
		Macro			Micro			Macro			Micro		
		P	R	F1	P	R	F1*	P	R	F1	P	R	F1
Together	Llama 3	**74.2**	38.6	47.3	65.3	32.8	43.7	**89.2**	32.4	42.2	83.7	22.9	35.9
	Llama 4	67.0	47.5	53.1	62.4	41.8	50.0	81.1	39.0	48.8	79.1	28.8	42.2
	Mixtral	61.2	39.0	44.2	58.2	33.3	42.4	77.4	34.4	43.2	77.7	24.1	36.8
Answer First	Llama 3	71.5	45.7	51.2	59.5	39.8	47.7	85.4	37.2	46.2	77.7	28.2	41.4
	Llama 4	66.8	51.3	**55.0**	56.9	**47.6**	**51.8**	84.7	43.7	**53.6**	79.1	35.9	**49.4**
	Mixtral	60.3	**51.6**	52.6	49.7	45.9	47.7	77.9	**45.6**	53.4	71.7	**36.0**	47.9
Evidence First	Llama 3	69.8	35.3	43.9	**67.3**	29.0	40.6	87.2	30.1	39.6	**85.2**	20.0	32.4
	Llama 4	64.0	38.7	44.9	59.2	32.8	42.2	80.4	33.1	42.5	78.1	23.5	36.1
	Mixtral	55.1	38.5	41.7	61.5	32.1	42.2	71.2	34.7	41.4	80.2	22.7	35.4
Heuristic	–	60.1	44.0	47.8	59.0	38.0	46.2	81.8	39.8	47.6	80.7	28.2	41.8

All scores are reported as percentages. *Macro:* average per-case F1; *Micro:* aggregate true/false positives and negatives across all cases; *P:* Precision; *R:* Recall; *F1:* F Score; *Llama 3:* Llama 3.3 70B; *Llama 4:* Llama 4 17B 16E; *Mixtral:* Mixtral 8x22B. Best and second-best scores are **bolded** and underlined, respectively. Essential-only Micro F1 is considered *Overall Factuality*.

**Table 6:** Relevance metrics results from benchmarking experiments using Human Answers as reference.

Approach	Model	BLEU	ROUGE	SARI	BERTScore	MEDCON	AlignScore	Overall Relevance
Together	Llama 3	2.4	21.0	49.2	39.3	36.7	**47.0**	32.6
	Llama 4	5.5	22.7	49.2	40.2	38.3	35.6	31.9
	Mixtral	5.7	23.1	51.8	40.2	39.9	38.3	33.2
Answer First	Llama 3	3.4	22.7	51.0	40.4	38.9	41.5	33.0
	Llama 4	6.7	23.6	51.7	40.3	38.1	36.0	32.7
	Mixtral	**6.8**	**24.2**	53.1	**42.5**	**40.2**	35.8	**33.8**
Evidence First	Llama 3	1.7	20.0	49.1	37.4	31.3	43.6	30.5
	Llama 4	4.0	20.3	48.8	36.9	32.8	37.4	30.0
	Mixtral	4.9	22.6	52.9	38.7	39.0	39.8	33.0
Heuristic	—	6.7	22.1	**53.6**	30.4	35.0	34.0	30.3

*“Overall Relevance”* is the mean of all normalized relevance scores. All scores are reported as percentages. *Llama 3:* Llama 3.3 70B; *Llama 4:* Llama 4 17B 16E; *Mixtral:* Mixtral 8x22B. Best and second-best scores are **bolded** and underlined, respectively.

**Table 7:** Relevance metrics results from benchmarking experiments using Note Excerpt and Questions as reference.

Approach	Model	BLEU	ROUGE	SARI	BERTScore		MEDCON	AlignScore	Overall Relevance
Together	Llama 3	0.3	17.8	49.4		23.7	29.0	59.0	29.9
	Llama 4	1.8	25.1	49.0		28.5	36.9	54.7	32.7
	Mixtral	1.5	22.9	52.9		27.1	35.2	54.2	32.3
Answer First	Llama 3	0.4	19.2	51.2		24.7	30.0	58.1	30.6
	Llama 4	1.9	25.3	51.4		28.2	34.9	54.0	32.6
	Mixtral	1.6	24.9	54.2		28.9	37.9	52.9	33.4
Evidence First	Llama 3	0.2	16.2	49.1		22.0	26.0	56.9	28.4
	Llama 4	1.1	22.0	48.6		25.6	32.5	55.9	31.0
	Mixtral	1.4	22.7	53.0		26.5	34.5	54.3	32.1
Heuristic	—	**11.7**	**37.3**	**72.3**		**38.8**	**44.1**	**66.1**	**45.0**

*“Overall Relevance”* is the mean of all normalized relevance scores. All scores are reported as percentages. *Llama 3:* Llama 3.3 70B; *Llama 4:* Llama 4 17B 16E; *Mixtral:* Mixtral 8x22B. Best and second-best scores are **bolded** and underlined, respectively.

**Table 8:** Overall scores from benchmarking experiments.

Approach	Model	Overall Score (Human)	Overall Score (Notes)
Together	Llama 3	38.1	36.8
	Llama 4	41.0	41.3
	Mixtral	37.8	37.3
Answer First	Llama 3	40.3	39.2
	Llama 4	**42.3**	42.2
	Mixtral	40.7	40.6
Evidence First	Llama 3	35.5	34.5
	Llama 4	36.1	36.6
	Mixtral	37.6	37.1
Heuristic	—	38.2	**45.6**

*“Overall Score”* is the average of *“Overall Relevance”* and *“Overall Factuality”* (Essential-only Micro F1). All scores are reported as percentages. *Human:* computed with relevance metrics using Human Answers from Table 6; *Notes:* computed with relevance metrics using Note Excerpt and Questions from Table 7; *Llama 3:* Llama 3.3 70B; *Llama 4:* Llama 4 17B 16E; *Mixtral:* Mixtral 8x22B. Best and second-best scores are **bolded** and underlined, respectively.

On *Factuality* (Table 5), when the evaluation considered the supplementary sentences as important, F1 scores dropped (driven primarily by a drop in Recall despite gains in Precision). This indicates that models were more effective at identifying the most important information from the clinical notes but struggled to consistently capture supporting details. Notably, macro F1 scores consistently exceeded the micro F1 scores, reflecting consistent performance across cases, despite variations in instance-level scores. Interestingly, *Together* approach often yielded higher precision, though at the expense of a lower recall, leading to modest F1 scores. Also, *Evidence First* consistently produced the poorest F1 scores, even underperforming the Heuristic approach, highlighting that constraining the evidence for the model creates a bottleneck for model performance.

On *Relevance* using human-authored answers (Table 6), *Evidence First* also underperformed across most metrics. This suggests that pre-selecting evidence negatively affects not only factual correctness but also the relevance to clinician answers. Among models, *Mixtral* attained the highest MEDCON score (40.2%, using *Answer First* approach), indicating strong alignment with clinically relevant medical concepts. Llama 3 attained the highest AlignScore (47.0%, using *Together*), underlining its superior alignment with the clinician answers despite modest performance on other metrics. The *Heuristic* approach showed consistently lower performance across metrics. Notably, its BERTScore dropped to 30.4%, significantly lower than the worst-performing model-based approach (36.9% using *Evidence First* and Llama 4).

When the generated answers were compared to the essential note sentences and the question (Table 7), the heuristic version achieved the highest scores—an artifact of the relevance metrics’ bias toward lexical overlap. Interestingly, the LLM-based variations achieved comparable scores using human answers or notes (Table 8). There is a statistically significant positive correlation between the overall relevance scores calculated using human answers and notes (Kendall’s *τ* = 0.362, p < 0.0001, n = 1000; comparing 100 individual case scores across 10 model variations), indicating moderate agreement.

#### Error analysis

During our manual error analysis of the highest and lowest scoring runs (based on Factuality and Relevance metrics) by the Llama 4 model using the Answer First approach, we observed that the automated metrics generally aligned with the actual quality of the generated responses. In most instances, the model-generated answers successfully captured the core content of the clinician-authored reference answers. For example, in one case, the model correctly inferred that the patient *“was given antibiotics because she developed pneumonia,”* but incorrectly attributed this decision to *“a chest x-ray that indicated low oxygen levels”*—a detail not supported by the clinical note and inconsistent with general medical knowledge. In another case, the model failed to mention an important related procedure (*“thoracentesis”*), despite its relevance to the question.

In one of the lowest-performing cases, the model incorrectly asserted that *“no specific order [was] mentioned for taking the prescribed medications,”* whereas the clinical note clearly included instructions for a *“dexamethasone taper.”* Moreover, in the same instance, the model disproportionately reproduced much of the content from patient’s question, rather than grounding the answer in clinical evidence. Interestingly, in some cases, although the model included citations to relevant sentences from the note, it failed to accurately convey the associated information in the generated text. Complete examples and corresponding annotations from our manual review are included in Table 9.

**Table 9:** System-generated responses by Llama 4 using the Answer First approach on the two best and worst performing cases on Overall Score.

Case ID: 23 (best factuality)	
**Patient Question**	Hi Dr. My mom is 88 years old, no heart problems. She hit her head and was hospitalized. No intubation was done in ICU. Her blood count and pressure were normal, she could eat and was breathing on her own. My question is about the antibiotics she was given during her hospital stay. Were they necessary? Thank you,
**Clinician Question**	Why was she given antibiotics during her hospital stay?
**Clinician Answer**	The patient was given antibiotics during her hospital stay because of a lung infection [5]. The patient was noted to have low levels of oxygen and a chest x-ray was ordered [5]. It indicated the patient was developing pneumonia [5]. IV antibiotics were initiated to treat the pneumonia [6]. The patient showed improvement right away after starting the antibiotics [6]. Therefore, the antibiotics were necessary during her hospital stay.
**System Answer**	```answer. Your mom was given antibiotics because she developed pneumonia, as shown by a chest x-ray that indicated low oxygen levels |5,6|. The antibiotics were started immediately and she showed improvement |6|.```
**Manual Review**	• Answers the question? ○ Yes: The antibiotics were initiated because of pneumonia. • Uses the evidence? ○ Supported: The antibiotics were initiated because of pneumonia. ○ Refuted: The chest x-ray was ordered due to low oxygen levels, not the other way around. • Uses general knowledge? ○ Conflicting: A chest x-ray does not measure oxygen saturation. • Formatting ○ Uses additional “answer” markdown block in the response.
**Case ID: 39 (best relevance)**	
**Patient Question**	hello, all doctors i am here for my mom is suffering from liver cirrhosis and stone in gall bladder, a few days back she felt unconcious then we took her to hospitol then she was kept in ICU for 10 days then after treatment she came back to censes, in the mean process aprox 1 and half litre water pumped from chast and now again there is sign of swelling and filling of water in there then again we consult our doctor they suggested for liver transplant,here now I am asking from you people that what is the best and proper way to cure her = .
**Clinician Question**	Is she getting any treatment for the swelling?
**Clinician Answer**	The patient was found to have a pleural effusion caused by liver disease [7]. The treatment she received for the swelling consisted of thoracentesis to fix the fluid accumulation [8]. The patient will continue on diuretics, lasix 10 mg daily and spirolactone 50 mg daily to help prevent a reoccurrence of the pleural effusion [9,14]. The patient will continue to follow up with Hepatology as indicated [16]. Her urine output and weight will be monitored and adjusted as needed [15].
**System Answer**	She is currently on diuretics, specifically lasix and spironolactone, to help prevent a reoccurrence of the pleural effusion |9|. Her diuretics were briefly held but have been restarted at lasix 10 mg daily and spironolactone 50 mg daily |13,14|. Her urine output and weight will continue to be monitored and adjusted as needed |15|.
**Manual Review**	• Answers the question? ○ Partially: It accurately describes the current medication regimen (diuretics), mentions the specific drugs and dosages, and notes the monitoring of urine output and weight. However, it does not mention thoracentesis and the plan to follow up with Hepatology. • Uses the evidence? ○ Supported: Uses appropriate evidence for the included clinical information. • Uses general knowledge? ○ No: The system does not incorporate additional general knowledge beyond what is included in the input note.
**Case ID: 118 (least relevance)**	
**Patient Question**	patient has been operated because of intestine, patient had scepticimia and surgery again for cleaning inside, what are the chances of survival and what can be the consequences after all these operations??
**Clinician Question**	What is the expected course of recovery after surgery and septicemia?
**Clinician Answer**	The patient is expected to continue on antibiotics via her PICC line when she is discharged, due to GNRs and GPCs that were present in the blood cultures [18,19,23,36]. Surgery included hysterectomy, salpingo-oopherectomy and omentectomy, resulting in a colostomy [9,23,33]. The patient will have an extended recovery as her abdominal wounds heal, and as she learns how to manage her colostomy [26,34,35]. The patient will be discharged to a rehab facility for extended recovery [24].
**System Answer**	```answer. The patient’s condition improved at discharge, with stable vital signs and decreasing white blood cell count |26,35|. She tolerated a regular diet, ambulated with assistance, and had well-controlled pain |26|. Expected course of recovery involves continued improvement with rehabilitation |24,25|. Close monitoring and management of potential complications, such as infection, will be necessary |18,21,23|. Underlying conditions like DM2, HTN, and HL require ongoing management |2|.```
**Manual Review**	• Answers the question? ○ Partially: The system output presents a general summary of the patient’s discharge condition and some aspects of the expected recovery. However, it fails to mention the continuation of antibiotics due to GNRs/GPCs, colostomy care, and wound healing. • Uses the evidence? ○ No: The citations used for infection management (|18,21,23|) are not explicitly tied to the continuation of antibiotics. • Uses general knowledge? ○ Yes: Uses general medical knowledge in mentioning the need for monitoring complications like infection and managing chronic conditions such as DM2, HTN, and HL. • Formatting ○ Uses additional “answer” markdown block in the response.
**Case ID: 99 (least factuality)**	
**Patient Question**	Hi Doc my husband has been identified with tuburculos meningitis as his protien was 496 / lympocytes 90% after lumber tap. he spent 5 days in ICU & now he is on akt 4 treatment. 01 Large Tablet after breakfast / rifamicin after 02 hours of breakfast and rest two after lunch is this the right way to take the medicines as earlier he was taking rifamicin empty stomach but this creates nausea and vomiting so our doc changed to the sequel mentioned. Also he has a fever around 100-101 please advise
**Clinician Question**	Is there a specified order to take the prescribed medications?
**Clinician Answer**	Patient was diagnosed with tuberculous meningitis and was discharged home on a long list of important medications that must be taken exactly as directed [2,3]. The dexamethasone taper medication has a specific order in which it is to be taken [21]. Patient is to start at 6 mg IV every 6 hours and decrease the dose over a period of time until transitioned to an oral dose [21]. This dose starts at 4 mg daily and gradually decreases until finished [21].
**System Answer**	There is no specific order mentioned for taking the prescribed medications |4,6,9,10,11|. However, the patient is currently taking one large tablet (likely Isoniazid) after breakfast, Rifampin after 2 hours of breakfast, and the rest two tablets after lunch. The current regimen seems to be adjusted to mitigate nausea and vomiting caused by Rifampin taken on an empty stomach. It is recommended to consult the doctor for further clarification on the medication schedule |24,27,28|.
**Manual Review**	• Answers the question? ○ No: It states that there is no specific order mentioned, which is incorrect. It fails to address the question regarding the specific order of taking the prescribed medications, particularly the dexamethasone taper. • Uses the evidence? ○ Refutes: It misrepresents the evidence by claiming that no specific order is mentioned. Further, it focuses on observed patient behavior (timing of pill intake from the question) rather than the prescribed regimen described in the evidence. • Uses general knowledge? ○ No: The system does not incorporate additional general knowledge beyond what is included in the input question and the note.

Note Excerpts are not included because of brevity and MIMIC data use agreement.

Our evaluation framework targeted two key dimensions: *Factuality*, assessing the cited note sentences, and *Relevance*, evaluating the answer text consistency with the reference response. Manual inspection showed alignment with these automated assessments. Nonetheless, we acknowledge the potential for more scalable and nuanced evaluations, such as leveraging advanced LLMs to assess answer quality, particularly in relation to annotated reference answers^48^.

### External validation

In addition to the rigorous validation protocol employed throughout the development of ArchEHR-QA, the dataset has also been subjected to community-based evaluation through a shared task (also titled ArchEHR-QA), organized as part of the BioNLP (Biomedical Natural Language Processing) Workshop at the 2025 Annual Meeting of the Association for Computational Linguistics (ACL 2025). This shared task invited researchers to explore and develop innovative methods for grounded EHR QA using the dataset. A total of 76 successful system submissions from 29 participating teams were recorded, further demonstrating the usability, relevance, and community interest in ArchEHR-QA as a benchmark resource.

Most participating teams in the shared task adopted a two-stage framework, where the identification of relevant clinical note sentences is followed by answer generation. Some top-scoring frameworks also performed answer reformulation or citation assignment post answer generation. All submitted systems used LLMs, proprietary (e.g., OpenAI’s ChatGPT, Google’s Gemini) or open-weight (e.g., Meta’s LLaMa, Mistral AI’s Mistral), while some also incorporated small language models (e.g., BERT) in their framework. There were few investigations into model fine-tuning strategies such as fine-tuning, few-shot learning, hyperparameter tuning, and the use of synthetic data. Also, some of the submitted systems post-processed model-generated responses using heuristics or the model itself.

### Limitations

The forum posts and MIMIC EHR notes originate from different patient populations. Nonetheless, the informational needs expressed in forum posts remain relevant and have been examined in contexts such as Radiology^49^ and Laboratory Results^50,51^. Additionally, no forum post was aligned with a note in a way that could alter its underlying information needs. Patient portal messages linked to EHRs could offer real-world data to study patient information needs. However, although studies using portal messages exist, the corresponding datasets are not publicly available^16,17,52,53^. Our approximate but methodical alignment provides a publicly accessible resource for advancing patient-specific EHR QA.

The developed dataset originates from a single medical institution and a single health discussion forum. However, the underlying EHR data are drawn from two distinct clinical settings (ICU and ED) and were authored by different providers over several years. The associated patient questions also reflect authentic information needs expressed in real-world contexts. Moreover, our proposed paradigm for aligning forum posts with corresponding EHRs is scalable and adaptable to a variety of EHR systems, clinical documentation formats, and health discussion forums.

## Usage Notes

The primary focus of studies on generating responses to patient portal questions has been on evaluating clinicians’ perception rather than evaluating the use of EHR information^21,22^. The proposed dataset addresses this gap by providing detailed sentence-level EHR note annotations, enabling fine-grained assessments of how well automated systems ground their responses in relevant clinical information. Additionally, there is growing evidence supporting the use of AI technologies to generate draft responses for clinician review^21,22,54^. This dataset facilitates the development of automated benchmarking protocols, accelerating system improvements, and ultimately helping to reduce clinician burden.

While the models are benchmarked, and demonstrate usability of the data, on the task of grounded EHR QA, the proposed dataset offers broader utility beyond this specific application. For instance, the annotated note excerpts can support the development and evaluation of clinical information retrieval systems^55^. Additionally, the clinician- authored questions represent a valuable resource for advancing research on summarizing patient questions^56^. Finally, the grounded QA task itself can also be extended to full-length clinical notes^57^, whose document identifiers are preserved and released with the dataset. Automated approaches to process long clinical documents may include retrieval-augmented generation^58^ (which combines information retrieval and generative models) and summarization techniques^59^ (that distill essential information while maintaining critical details) among other advanced natural language processing methods.

## Supplementary information

Supplementary Materials

## Data Availability

The ArchEHR-QA dataset is publicly available via PhysioNet^36^ at 10.13026/zzax-sy62.

## Code availability

The accompanying code used to evaluate the baseline systems is available via GitHub at https://github.com/soni-sarvesh/archehr-qa/tree/main/evaluation.

## References

[1] Ely, J. W., Osheroff, J. A., Chambliss, M. L., Ebell, M. H. & Rosenbaum, M. E. Answering Physicians’ Clinical Questions: Obstacles and Potential Solutions. *J. Am. Med. Inform. Assoc.* **12**, 217–224 (2005). 15561792 10.1197/jamia.M1608 PMC551553

[2] Bardhan, J., Roberts, K. & Wang, D. Z. Question Answering for Electronic Health Records: Scoping Review of Datasets and Models. *J. Med. Internet Res.* **26**, e53636 (2024). 39475821 10.2196/53636 PMC11561445

[3] Soni, S., Datta, S. & Roberts, K. quEHRy: a question answering system to query electronic health records. *J. Am. Med. Inform. Assoc.* **30**, 1091–1102 (2023). 37087111 10.1093/jamia/ocad050 PMC10198534

[4] Fisher, B., Bhavnani, V. & Winfield, M. How patients use access to their full health records: A qualitative study of patients in general practice. *J. R. Soc. Med.* **102**, 538–544 (2009). 10.1258/jrsm.2009.090328 PMC2789021 19966130

[5] Woods, S. S. *et al*. Patient experiences with full electronic access to health records and clinical notes through the my healthevet personal health record pilot: Qualitative study. *J. Med. Internet Res.* **15**, 403 (2013). 10.2196/jmir.2356 PMC3636169 23535584

[6] Davis Giardina, T., Menon, S., Parrish, D. E., Sittig, D. F. & Singh, H. Patient access to medical records and healthcare outcomes: a systematic review. *J. Am. Med. Inform. Assoc.* **21**, 737–741 (2014). 24154835 10.1136/amiajnl-2013-002239 PMC4078277

[7] Tapuria, A. *et al*. Impact of patient access to their electronic health record: systematic review. *Inform. Health Soc. Care* **46**, 194–206 (2021). 10.1080/17538157.2021.1879810 33840342

[8] Bell, S. K. *et al*. Do patients who read visit notes on the patient portal have a higher rate of “loop closure” on diagnostic tests and referrals in primary care? A retrospective cohort study. *J. Am. Med. Inform. Assoc*. ocad250, 10.1093/jamia/ocad250 (2024). 10.1093/jamia/ocad250 PMC10873783 38164964

[9] Pieper, B. *et al*. Discharge Information Needs of Patients After Surgery. *J. Wound. Ostomy Continence Nurs.* **33**, 281 (2006). 16717518 10.1097/00152192-200605000-00009

[10] Zavala, S. & Shaffer, C. Do Patients Understand Discharge Instructions? *J. Emerg. Nurs.* **37**, 138–140 (2011). 21397126 10.1016/j.jen.2009.11.008

[11] Arora, A. *et al*. The value of standards for health datasets in artificial intelligence-based applications. *Nat. Med.* **29**, 2929–2938 (2023). 37884627 10.1038/s41591-023-02608-w PMC10667100

[12] Welivita, A. & Pu, P. A survey of consumer health question answering systems. *AI Mag.* **44**, 482–507 (2023).

[13] Zhang, Y. & Fu, W.-T. Designing Consumer Health Information Systems: What Do User-Generated Questions Tell Us? in *Foundations of Augmented Cognition. Directing the Future of Adaptive Systems* (eds Schmorrow, D. D. & Fidopiastis, C. M.) 536–545, 10.1007/978-3-642-21852-1_62 (Springer, Berlin, Heidelberg, 2011).

[14] Cimino, J. J. Putting the “why” in “EHR”: capturing and coding clinical cognition. *J. Am. Med. Inform. Assoc.* **26**, 1379–1384 (2019). 31407781 10.1093/jamia/ocz125 PMC6798564

[15] Martinez, K. A., Schulte, R., Rothberg, M. B., Tang, M. C. & Pfoh, E. R. Patient Portal Message Volume and Time Spent on the EHR: an Observational Study of Primary Care Clinicians. *J. Gen. Intern. Med.* **39**, 566–572 (2024). 38129617 10.1007/s11606-023-08577-7 PMC10973312

[16] Liu, S. *et al*. Leveraging large language models for generating responses to patient messages—a subjective analysis. *J. Am. Med. Inform. Assoc*. ocae052, 10.1093/jamia/ocae052 (2024). 10.1093/jamia/ocae052 PMC11105129 38497958

[17] Biro, J. M. *et al*. Opportunities and risks of artificial intelligence in patient portal messaging in primary care. *Npj Digit. Med.* **8**, 1–6 (2025). 40275104 10.1038/s41746-025-01586-2 PMC12022076

[18] Kaur, A., Budko, A., Liu, K., Steitz, B. D. & Johnson, K. B. Primary Care Providers Acceptance of Generative AI Responses to Patient Portal Messages. *Appl. Clin. Inform*. **0** (2025). 10.1055/a-2565-9155 PMC12310298 40132987

[19] Baxter, S. L., Longhurst, C. A., Millen, M., Sitapati, A. M. & Tai-Seale, M. Generative artificial intelligence responses to patient messages in the electronic health record: early lessons learned. *JAMIA Open* **7**, ooae028 (2024). 38601475 10.1093/jamiaopen/ooae028 PMC11006101

[20] Chen, S. *et al*. The effect of using a large language model to respond to patient messages. *Lancet Digit. Health* **0** (2024). 10.1016/S2589-7500(24)00060-8 PMC11829255 38664108

[21] Small, W. R. *et al*. Large Language Model–Based Responses to Patients’ In-Basket Messages. *JAMA Netw. Open* **7**, e2422399 (2024). 39012633 10.1001/jamanetworkopen.2024.22399 PMC11252893

[22] Garcia, P. *et al*. Artificial Intelligence–Generated Draft Replies to Patient Inbox Messages. *JAMA Netw. Open* **7**, e243201 (2024). 38506805 10.1001/jamanetworkopen.2024.3201 PMC10955355

[23] Haug, C. J. & Drazen, J. M. Artificial Intelligence and Machine Learning in Clinical Medicine, 2023. *N. Engl. J. Med.* **388**, 1201–1208 (2023). 36988595 10.1056/NEJMra2302038

[24] Shah, N. H., Entwistle, D. & Pfeffer, M. A. Creation and Adoption of Large Language Models in Medicine. *JAMA*, 10.1001/jama.2023.14217 (2023). 10.1001/jama.2023.14217 37548965

[25] Im, E.-O. & Chee, W. An Online Forum As a Qualitative Research Method: Practical Issues. *Nurs. Res.* **55**, 267–273 (2006). 16849979 10.1097/00006199-200607000-00007 PMC2491331

[26] Seale, C., Charteris-Black, J., MacFarlane, A. & McPherson, A. Interviews and internet forums: a comparison of two sources of qualitative data. *Qual. Health Res.* **20**, 595–606 (2010). 20008955 10.1177/1049732309354094

[27] Li, Y. *et al*. ChatDoctor: A Medical Chat Model Fine-Tuned on a Large Language Model Meta-AI (LLaMA) Using Medical Domain Knowledge. *Cureus* **15** (2023). 10.7759/cureus.40895 PMC10364849 37492832

[28] Johnson, A. E. W. *et al*. MIMIC-III, a freely accessible critical care database. *Sci. Data* **3**, 160035 (2016). 27219127 10.1038/sdata.2016.35 PMC4878278

[29] Johnson, A. E. W. *et al*. MIMIC-IV, a freely accessible electronic health record dataset. *Sci. Data* **10**, 1 (2023). 36596836 10.1038/s41597-022-01899-x PMC9810617

[30] Johnson, A., Pollard, T. & Mark, R. MIMIC-III Clinical Database. *PhysioNet*, 10.13026/C2XW26 (2015).

[31] Johnson, A., Pollard, T., Horng, S., Celi, L. A. & Mark, R. MIMIC-IV-Note: Deidentified free-text clinical notes. *PhysioNet*, 10.13026/1N74-NE17 (2023).

[32] Bell, R. A., Hu, X., Orrange, S. E. & Kravitz, R. L. Lingering questions and doubts: online information-seeking of support forum members following their medical visits. *Patient Educ. Couns.* **85**, 525–8 (2011). 21315538 10.1016/j.pec.2011.01.015

[33] Grattafiori, A. *et al*. The Llama 3 Herd of Models. Preprint at, 10.48550/arXiv.2407.21783 (2024).

[34] Robertson, S. E., Walker, S., Jones, S., Hancock-Beaulieu, M. & Gatford, M. Okapi at TREC-3. in *Proceedings of the 3rd Text REtrieval Conference* 109–126 (1994).

[35] Soni, S. & Demner-Fushman, D. ArchEHR-QA: BioNLP at ACL 2025 Shared Task on Grounded Electronic Health Record Question Answering. *PhysioNet*, 10.13026/zzax-sy62 (2025).

[36] Goldberger, A. L. *et al*. PhysioBank, PhysioToolkit, and PhysioNet. *Circulation* **101**, e215–e220 (2000). 10851218 10.1161/01.cir.101.23.e215

[37] Lin, J. *et al*. What Makes a Good Answer? The Role of Context in Question Answering. in *Proceedings of the Ninth IFIP TC13 International Conference on Human-Computer Interaction* (Zurich, Switzerland, 2003).

[38] Jeon, J., Croft, W. B., Lee, J. H. & Park, S. A framework to predict the quality of answers with non-textual features. in *Proceedings of the 29th annual international ACM SIGIR conference on Research and development in information retrieval* 228–235, 10.1145/1148170.1148212 (Association for Computing Machinery, New York, NY, USA, 2006).

[39] Wang, W. *et al*. MiniLM: Deep Self-Attention Distillation for Task-Agnostic Compression of Pre-Trained Transformers. in *Advances in Neural Information Processing Systems* vol. 33 5776–5788 (Curran Associates, Inc., 2020).

[40] The Llama 4 herd: The beginning of a new era of natively multimodal AI innovation. *Meta AI* https://ai.meta.com/blog/llama-4-multimodal-intelligence/.

[41] Jiang, A. Q. *et al*. Mixtral of Experts. Preprint at 10.48550/arXiv.2401.04088 (2024).

[42] Papineni, K., Roukos, S., Ward, T. & Zhu, W.-J. BLEU: a method for automatic evaluation of machine translation. in *Proceedings of the 40th annual meeting on association for computational linguistics* 311–318, 10.3115/1073083.1073135 (Association for Computational Linguistics, 2002).

[43] Lin, C.-Y. ROUGE: A Package for Automatic Evaluation of Summaries. in *Text Summarization Branches Out* 74–81 (Association for Computational Linguistics, Barcelona, Spain, 2004).

[44] Xu, W., Napoles, C., Pavlick, E., Chen, Q. & Callison-Burch, C. Optimizing Statistical Machine Translation for Text Simplification. *Trans. Assoc. Comput. Linguist.* **4**, 401–415 (2016).

[45] Zhang, T., Kishore, V., Wu, F., Weinberger, K. Q. & Artzi, Y. BERTScore: Evaluating Text Generation with BERT. in *International Conference on Learning Representations* (2019).

[46] Zha, Y., Yang, Y., Li, R. & Hu, Z. AlignScore: Evaluating Factual Consistency with A Unified Alignment Function. in *Proceedings of the 61st Annual Meeting of the Association for Computational Linguistics (Volume 1: Long Papers)* (eds Rogers, A., Boyd-Graber, J. & Okazaki, N.) 11328–11348, 10.18653/v1/2023.acl-long.634 (Association for Computational Linguistics, Toronto, Canada, 2023).

[47] Yim, W. *et al*. Aci-bench: a Novel Ambient Clinical Intelligence Dataset for Benchmarking Automatic Visit Note Generation. *Sci. Data* **10**, 586 (2023). 37673893 10.1038/s41597-023-02487-3 PMC10482860

[48] Gao, M. *et al*. LLM-based NLG Evaluation: Current Status and Challenges. *Comput. Linguist*. 1–28, 10.1162/coli_a_00561 (2025).

[49] Alarifi, M., Patrick, T., Jabour, A., Wu, M. & Luo, J. Understanding patient needs and gaps in radiology reports through online discussion forum analysis. *Insights Imaging* **12**, 50 (2021). 33871753 10.1186/s13244-020-00930-2 PMC8055745

[50] Reynolds, T. L. *et al*. Understanding Patient Questions about their Medical Records in an Online Health Forum: Opportunity for Patient Portal Design. in *AMIA Annual Symposium Proceedings* 1468–1477 (2017). PMC5977702 29854216

[51] Reynolds, T. L., Zhang, J., Zheng, K. & Chen, Y. Unpacking the Use of Laboratory Test Results in an Online Health Community throughout the Medical Care Trajectory. in *Proceedings of the ACM on Human-Computer Interaction* vol. 6 361:1-361:32 (2022).

[52] Anderson, B. J. *et al*. Development and Evaluation of a Model to Manage Patient Portal Messages. *NEJM AI* **2**, AIoa2400354 (2025).

[53] Liu, S. *et al*. Detecting emergencies in patient portal messages using large language models and knowledge graph-based retrieval-augmented generation. *J. Am. Med. Inform. Assoc.* **32**, 1032–1039 (2025). 40220286 10.1093/jamia/ocaf059 PMC12089757

[54] Reynolds, K. & Tejasvi, T. Potential Use of ChatGPT in Responding to Patient Questions and Creating Patient Resources. *JMIR Dermatol.* **7**, e48451 (2024). 38446541 10.2196/48451 PMC10955382

[55] Sivarajkumar, S. *et al*. Clinical Information Retrieval: A Literature Review. *J. Healthc. Inform. Res.* **8**, 313–352 (2024). 38681755 10.1007/s41666-024-00159-4 PMC11052968

[56] Ben Abacha, A. *et al*. Overview of the MEDIQA 2021 Shared Task on Summarization in the Medical Domain. in *Proceedings of the 20th Workshop on Biomedical Language Processing* 74–85, 10.18653/v1/2021.bionlp-1.8 (Association for Computational Linguistics, Online, 2021).

[57] Adams, L. *et al*. LongHealth: A Question Answering Benchmark with Long Clinical Documents. *J. Healthc. Inform. Res.* **9**, 280–296 (2025). 40726742 10.1007/s41666-025-00204-w PMC12290132

[58] Lewis, P. *et al*. Retrieval-augmented generation for knowledge-intensive NLP tasks. in *Advances in neural information processing systems* (eds Larochelle, H., Ranzato, M., Hadsell, R., Balcan, M. F. & Lin, H.) vol. 33 9459–9474 (Curran Associates, Inc., 2020).

[59] Laban, P., Fabbri, A., Xiong, C. & Wu, C.-S. Summary of a Haystack: A Challenge to Long-Context LLMs and RAG Systems. in *Proceedings of the 2024 Conference on Empirical Methods in Natural Language Processing* (eds Al-Onaizan, Y., Bansal, M. & Chen, Y.-N.) 9885–9903, 10.18653/v1/2024.emnlp-main.552 (Association for Computational Linguistics, Miami, Florida, USA, 2024).

