# Supplementary material for: A Dataset for Addressing Patient’s Information Needs related to Clinical Course of Hospitalization
Source: Sci Data. 2026 Feb 25;13:523. doi: 10.1038/s41597-026-06639-z (PMC13046737; doi:10.1038/s41597-026-06639-z)
Supplement: Supplementary file 1 — Supplementary Materials [file 41597_2026_6639_MOESM1_ESM.pdf]

Supplementary Materials for “A Dataset for Addressing Patient’s Information Needs related to Clinical Course of Hospitalization”

Annotation Guidelines

1. Alignment

You will be provided with a patient-posed question from a public health discussion forum and a set of 10 candidate discharge summaries or notes, selected based on lexical and semantic similarity. Your task has two components:

(a) Note Selection for Alignment

- Select the discharge summary that is most appropriate for answering the question.
- The selected note should not contradict the question. While a perfect match is not expected, the alignment must be contextually plausible.
- Avoid selecting discharge summaries with mismatched core clinical facts.
  - Example: If the question is about the reason for a major procedure (e.g., CABG), and the note does not mention such a procedure, it is not a suitable match.

(b) Question Editing

- Edit the patient-posed question to reflect the patient characteristics and clinical context in the selected discharge summary.
- Make only the minimal changes required to ensure consistency between the question and the selected note without altering the core information need.
  - Example: If the question references a 56-year-old, but the note refers to a 58-year-old with otherwise matching context, change the age to 58 in the edited question.
- Do not add new clinical inquiries or modify the underlying intent of the question.
- Note: the clinical document is read only; it cannot be modified.

2. Clinician Question Creation and Sentence Relevance Annotations

Given an edited patient-posed question and its associated discharge summary, you will complete two sub-tasks:

(a) Clinician Question Formulation

- Identify the main information needs embedded in the patient's question.

- Reformulate it from the clinician's perspective to produce a clear and focused Clinician Question.
- This question should express how a clinician would interpret the patient's question and respond to it.
- Additionally, annotate the focus areas in the patient's question that prompted the Clinician Question.

(b) Sentence Relevance Annotation

- The discharge summary is pre-split into individual sentences.
- For each sentence, assign one of the following relevance labels indicating its importance in answering the Clinician Question:
    - Essential: Provides critical and necessary information to directly answer the Clinician Question. Note: if several sentences contain the same critical information, all should be labeled as essential.
    - Supplementary: Offers supporting or contextual information but is not strictly required.
    - Not-Relevant: Does not contribute meaningfully to answering the question. This is the default label unless updated.

3. Answer Creation

Given the patient-posed question with focus areas, the clinician-interpreted question, and the annotated discharge summary with sentence-level relevance annotations, you will compose an answer. The following are the guidelines to create an answer:

- Write a succinct answer (approximately 75 words or 5 sentences) that directly addresses the Clinician Question.
- The answer must be fact-based and entirely grounded in the discharge summary.
    - Do not introduce or infer information that is not explicitly stated in the discharge summary.
- All Essential sentences must be cited in your answer using bracketed numeric references (e.g., “[1]”, “[2,3]”).
    - Supplementary sentences may be cited for clarity or completeness but are not required.
- Ensure that the answer:
    - Fully addresses all aspects of the Clinician Question.
    - Is concise, medically accurate, and free of speculation.
    - Is written in a neutral, informative tone, suitable for a clinical audience (layperson language is not required).
